# Surgical and Pathological Observations on the Eye

**Published:** 1840

**Authors:** William A. McDowell

**Affiliations:** Louisville, Kentucky


### Art. II.—*Surgical and Pathological Observations on the Eye.* By William A. McDowell, M. D., of Louisville, Kentucky.

*Note.*—In making the following communication, my chief objects are to make known a supposed discovery in the Anatomy of the Eye, and to give publicity to an operation, the *Section of the Conjunctiva,* which is believed to constitute an improvement in the surgical treatment of conjunctivitis. This I had at first designed to accomplish by appending cases operated upon to an article on the Surgical and Pathological Anatomy of the Eye; but, in reporting cases, it is necessary to give a summary of treatment, which, in some of my cases, was so discrepant from that of the standard authorities of the day, that I conceived it due to myself to assign my reasons for the difference. This I found I could not render intelligible, or satisfactory to the reader, without going into a demonstration of the adaptation of my therapeutics to the pathology in the cases, which would give to them an unwarrantable prolixity. To accomplish all of these objects, the present seemed to me the most eligible form, and if my efforts at condensation, to adapt such an essay to the limits of a periodical, shall not too far have sacrificed perspicuity to brevity, I trust it will prove the most acceptable to the profession.

---

In the following observations on the surgical and pathological Anatomy of the Eye, I shall mainly aim at the demonstration of two points in the structure of the conjunctiva, which I conceive to be of great surgical and pathological importance, but which seem to have been entirely overlooked by writers on the eye, both anatomical and surgical. These are, that the conjunctiva is double-coated; and that it is immediately and intimately connected with the ciliary processes, and through them with the internal structure of the eye.

The globe of the eye is made up of coats and humors. The coats proper, reckoning from without inwards, are, the sclerotica and cornea, the choroides and iris, the retina and lenticular capsule, and the tunica hyaloideia. These tunics are all either connected with, or arise from, the ciliary body, an elevated vascular ring, situated at the junction of the sclerotica and cornea; and are here intimately united with it, and with one another—constituting this, in a pathological point of view, a most interesting part, being a common point of union between all the important structures of the globe.

Thus far I am in accordance with all authorities on the subject, who have been minute in their examinations and descriptions of those structures. But to the conjunctiva, the internal tunic of the palpebræ, and the external coat of the anterior hemisphere of the eyeball, they appear to have devoted but little attention; although, from its exposed situation, its highly vascular and nervous structure, and from the great variety of injuries and diseases to which it is liable, it would seem to be of more surgical and pathological importance than any of the others. The conjunctiva is composed of *two laminæ, a mucous, and a fibrous,* which may be dissected apart with but little difficulty. In the eye of the ox they are separated with great facility. The outer, the mucous coat, is but little vascular, exhibits no red vessels even in high states of conjunctivitis, except when being covered with granulations, and they then appear tortuous and reticulated, as seen in other granulating surfaces. The inner, or fibrous, which is so nervous as to have rendered the tenderness of "the apple of the eye" proverbial, is also extremely vascular. Many of those vessels are seen filled with red blood, radiating towards the cornea in eyes in the healthy state; but in intense conjunctivitis, when red blood is thrown into the smaller ramifications, its vascularity is sometimes such as to exhibit the appearance of a ball of fire.

It is between these coats, and not between the conjunctiva and the sclerotica, as has been taught, that effusions occur in chemosis, separating and protruding a fold of the mucous coat over the cornea.

In the eyes of bullocks, that had been knocked on the head with an axe, I have met with highly injected, and with ruptured vessels and ecchymosis near the cornea; the consequence I presume of the concussion.

With the the point of a probe, I have separated the mucous coat and raised it off of the effusion, and washed it away—leaving the injected vessel entire, and embedded in the subjacent thicker fibrous coat. Raising this fibrous coat, brings to view (the tendinous expansions) the albuginia.

The conjunctiva is loosely attached by cellular tissue to the subjacent albuginia, and is easily separted from it with a probe, up to the margin of the cornea. Here the point of the probe meets with resistance from a connection so firm, that if forced forward, it ruptures and passes through the conjunctiva. But raise the conjunctiva to the cornea, and at the line of junction of the cornea with the sclerotica, divide with a knife a vertical fibrous structure, to the breadth of about a fourth of a line or less ; then push on the probe, and it will pass on under the membrane over the cornea, as easily, as it passed up to it, until it reaches the opposite side, where it again meets with resistance; but so much less, that if it is cautiously urged on, it may pass out from the cornea without rupturing the pellicle that invests it. On emerging, the probe is found between the mucous and fibrous coats of the conjunctiva. This vertical structure is believed to be constituted, at least in due proportion, of nerves and blood-vessels that perforate the coats at the junction of the cornea with the sclerotica, and intermingle and inosculate with the internal structures at the ciliary body. The following pathological facts in the nervous system favor this opinion.

Intolerance of light is one of the premonitory symptoms of conjunctivitis; it is found connected with the irritable, the nervous stage of the disease before any vascular congestion or inflammatory action is manifested; and when inflammation is fully developed and the consequent secretion of mucus or of pus supervenes and lessens the nervous irritation, intolerance of light diminishes or ceases. There is even some reason to beleve that the sensibility of the iris solely depends upon nervous fibres from this source:

1st. Because there would seem to be no need of sensitive nerves to any of the other inner structures.

2d. Because punctures of the retina and choroides made by oculists have been unattended by pain.

3d. Because diseases of internal structures, when confined to one eye, have gone on to perfect blindness, affecting the iris in the progress of disorganization, to the production of immobility, and even to the closure of the pupil, unaccompanied either by pain or intolerance of light; sometimes without the patient's knowledge that disease existed.*

4th. Because internal pain is speedily relieved by a complete division of the conjunctiva around the cornea.

The following phenomena in the vascular system support the opinion:

In moderate degrees of conjunctivitis, many vessels carrying red blood are seen coursing over the surface of the globe of the eye to the cornea, and there stop short abruptly. Others, in more severe cases, are seen to extend over the cornea, but the corneal extension is constantly much diminished in size, seldom equalling half the size of the same vessel without the circumference of the cornea. At the cornea, too, they present the appearance of an enlargement, such as is generally observed in other vessels at points of bifurcation. In vehement conjunctivitis we see the inflammatory action extending to and through the inner structures of the eye with great rapidity; in a few days, producing iritis, retinitis, cataract, and amaurosis; followed by rupture of the cornea, closure of the pupil, synechia anterior, and staphyloma.

This intercommunication—this inosculation—between the vessels of the conjunctiva and the internal structures, is still more strongly manifested in cases of primitive internal inflammation; as in iritis and retinitis, in which cases a red zone of vessels is seen all around the cornea, arising out of its line of junction with the sclerotica, and radiating divergently back, lessening as they proceed.

This zone is treated of, and its appearance accurately de-

---

* See Lawrence on the Eye pp. 233, 252.

scribed, by all the authorities that I have had an opportunity to consult; but they all, in every instance, locate it in the sclerotica. I have often met with this zone, in cases of internal inflammation, or incipient amaurosis. The congestion was always in the conjunctiva; and local bloodletting, by scarifying the vessels of this zone has appeared to me, in some cases, to have constituted a very efficient part of my treatment.

Mr. Lawrence, speaking of iritis, says, " there is more or less external redness of the eye in the form of a red band round the cornea, deeper colored in front, and gradually shaded off behind ;" and again, in the same paragraph, " the pink tint of the inflamed *sclerotica*, and of the trunks lying on it, which is obscured in all inflammations of the membrane, is probably owing to their being covered by, and consequently seen through the conjunctiva. These vessels advance in nearly straight lines from the *circumference of the globe*, ramifying toward the front and are lost in the pink zone. The redness of the *sclerotica*, and the distension of its trunks increase as the affection proceeds."

In such descriptions all the authorities that I have access to concur, and in terms so similar, that they seem almost to have copied one from another. I quote the words of Mr. Lawrence from among them, only because he is one of the most modern, and most eminent writers on the subject. They all likewise advert to the disorganization of the inner structures, from conjunctivitis pretty much as I have described them ; but all agree in first involving the *sclerotica*, and thence deriving the internal disease. Why they have done so I cannot tell, unless it was that they knew no other mode of bringing about the direct vascular intercommunication between the outer and inner structures, which they so palpably discovered to exist.

From the meagre supply of nerves and blood vessels to the sclerotica, I should suppose sclerotitis to be a most rare disease. I have never met with a case of it, that I am aware of, although I do not doubt its occasional occurrence. Others may have observed it, but I do not consider it at all probable that many persons, if any, have *ever seen* an inflamed sclerotica, except in a *post mortem* examination.

Writers in their descriptions of those vessels, allude to their pink color, as caused by their being seen through the conjunctiva. Now the sclerotica is not in contact with the conjunctiva. The tendinous, and less diaphonous albuginia intervenes, and a probe, passed under a muscle of the eye on towards the cornea, as far as its insertions near the cornea will admit, cannot be seen through these two membranes, even in their healthy and most diaphonous state, unless it be pressed against them so as to attenuate their structure. How then, in conjunctivitis, where the membrane is thickened, turbid, and covered with granulations, can we see a vessel in the sclerotica? I conceive it must have been the vessels in the fibrous coat of the conjunctiva, seen through the thickened mucous coat, that have deceived and misled the writers on this subject, the vessels appearing deeper, and becoming less distinct, and paler, as the granulations increased, and the mucous tissue thickened.

The settlement of this question of structure is important—pathologically, as demonstrating the *mode* of the already known internal connection, that exists between diseases of all the structures of the eye with conjunctivitis, in the relation either of cause or effect—therapeutically, as affording to the surgeon an accessible medium through which to operate upon internal diseases of the eye by external applications, and putting it fully in his power, in case of external disease of such intensity as to threaten important internal structures, to cut off communication, and arrest its progress by the *section of the conjunctiva.*

*Conjunctivitis.*—From inflammation of the conjunctiva nearly all the diseases incident to the eye and its appendages originate. Loss of vision is more frequently consequent upon diseases of this membrane as a primitive seat, than upon those of all the other structures of the eye taken together. Yet less attention seems to have been paid to its structure and connexions than to those of any of the others. Its attachment to the surface of the cornea, and its important inosculations around it with internal structures, and its double structure, have not been attended to, probably not looked for whilst much ability and research have been devoted to the division and separation into laminæ of internal structures that seem to me to be of immeasurably less importance.

To exhibit the pathological and surgical advantages that may be derived from closer attention to the structure and connexions of the conjunctiva, is the chief object of this essay.

This, I conceive, will be more readily apprehended from a consideration of some of its most prominent idiopathic diseases, and as this is the only mucous membrane that is connected with the globe of the eye, and in order that no misapprehension may arise about the locality of the primary disease, I shall restrict myself to the consideration of such disorders as are generally admitted to be incident exclusively to mucous membranes, viz: catarrhal, and purulent or gonorrhœal.

*Catarrhal conjunctivitis — Causes.*—Catarrhal opthalmia generally owes its origin to atmospheric vicissitudes, as sudden changes from hot to cold, from dry to moist, &c. The conjunctiva is alike subject, though not in an equal degree liable, to catarrhal affections, with other mucous membranes, as the schneiderian, tracheal, and bronchial. In influenzas, those membranes are sometimes all thrown into catarrhal inflammation at the same time.

*Symptoms.*—In considering the symptoms, the course of treatment to which I give a preference, both in catarrhal and purulent opthalmia, will render it expedient to consider them in three different aspects or stages, which I will denominate the irritable, the active inflammatory, and the passive inflammatory. As the terms irritation and inflammation, are by many writers employed as synonymous it will be best here to define the meaning I attach to them.

The irritable, is the spasmodic, or nervous period of inflammatory action, characterized by pain only, the part seeming paler rather than redder. The active inflammatory is the period of increased vascular action in the irritated part, and is the result of reactions from the spasmodic or irritable stage. It is characterized by pain, redness, heat, and swelling. The passive inflammatory stage, is the period of debility or exhaustion, arising from long continued, or excessive action and vascular distention. It is characterized by disorganization, or change of structure indicated by diminished pain, darker red, less heat, and more swelling.

The first or irritable stage is marked by a sense of dryness, stiffness, and a sensation as if particles of sand were between the palpebræ and the globe, and an unpleasant sense of tightness, with intolerance of light; but that which most distinctly marks the duration of this period, and which generally lasts from twelve to thirty-six hours, is the suspension of the secretion of mucus. The lachrymal gland, excited by the neighboring irritation, discharges frequent showers of tears over the unprotected surface, producing a scalding sensation, and increasing the irritation. These incipient symptoms go on increasing in intensity, until pain and intolerance of light become very severe, the irritation of distension becoming superadded to that of the primitive irritant, until the appearance of redness and congestion of the hitherto colorless membrane, indicates the commencement of the second stage.

Redness and congestion, the first indications of this stage, commence in the conjunctiva palpebræ, and progress more or less rapidly, according to the violence of the disease, until in most cases it extends quite over its orbicular expansion to the cornea, assuming, in its whole extent, a bright scarlet color; and, during the progress of this congestion, the secretion of mucus returns, and the weeping ceases. With the progress of mucous secretion, the pain and intolerance of light subside. During this stage, the redness becomes less by day, and the pain and intolerance of light remit and cease; towards evening, smarting, a sense of fulness, and increase of redness come on and increase through the fore part of the night, with diminished mucous and increased lachrymal secretion. Towards morning the exacerbation subsides, with a considerably increased flow of mucus.

The disease thus progresses, generally becoming milder in each succeeding paroxysm, and with proper treatment, and in mild cases sometimes even without treatment, terminates by resolution. It rarely goes beyond this stage, but sometimes, in bad cases, or in cases under mal-treatment, the discharge changes to muco-purulent, and the pain and intolerance of light increase, until diseased action is extended to the cornea, iris, and lens, as occurs in purulent opthalmia. This unwonted violence, or a neglected and protracted state of the active inflammatory stage, sometimes exhausts the contractility of the congested vessels, and relaxes them to such a degree that, when increased arterial action has subsided, they remain sluggish and relaxed, and are kept distended by the natural *vis a tergo*, and present the characteristics of the third or passive inflammatory stage. This is characterized by redness of the membrane approaching to crimson, chemosis, tumid palpebral margins, sometimes resulting in ektropion, suppuration and granulation of the surface of the conjunctiva, a nebulous cornea, internal disorganization and total blindness.

### TREATMENT.

The obvious objects of treatment in this disease are, first—to prevent the occurrence of inflammation, by interrupting irritation and by restoring functional secretion; second—to remove inflammation; third—to prevent or arrest disorganization.

*First stage.*—If consulted at an early period of the disease, before vascular congestion has become very evident, a full purgative dose of neutral salts, followed in two or three hours by full draughts of acidulated whey or panada, and by the application to the eyes of 20 or 30 drops of tinct. opii, will but rarely, if ever, fail to arrest the disease. If vascular congestion has somewhat advanced, and is accompanied by increased general arterial action, the eye being still in the stage of lachrymation and deficient mucous secretion, the above treatment should be premised by bloodletting. The salts and the hot drink arouse the secreting functions of the alimentary canal and of the skin; and the conjunctiva, being a muco-cutaneous membrane, and directly connected by continuity of surface, both with the mucous coat of the alimentary canal and the skin, is thus doubly stimulated by associated action to a resumption of its secretory functions. The purgative operation at the same time relieving congestion, by producing a tendency of the circulating blood from the head to the messentery; whilst the local application promotes the end, by acting at once as an anti-spasmodic and a powerful counter-irritant. In the prevalence of epidemic conjunctivitis, as in opthalmic influenza, I have enjoyed the opportunity of thus treating the disease in this stage, with the most perfectly satisfactory results.

*Second stage.*—In the treatment of this stage, too little importance, I conceive, has been given to the paroxysmal character of the inflammation. As marked advantage is derived here from the proper adaptation of remedies to the periods of remission and exacerbation, as from corresponding observations in the treatment of fevers.

The general treatment consists in bloodletting, as often repeated and to such extent as is requisite to keep general arterial action rather lower than the healthy standard, and in keeping the bowels duly open with saline purgatives. Diffusible stimulating diaphoretics should be freely used through the diurnal remission, such as vin. antimon. and spts. nit. dulc. or camphor and ammonia in small doses, along with copious drinks of acidulated hot whey or panada. In obstinate cases, one or two grains of opium and ten of pulv. Doveri, may be administered with great advantage two or three hours before the recurrence of the nocturnal exacerbation. During the exacerbation, administer antimonial diaphoretics every two or three hours, and cool diluent drinks.

*Local treatment.*—Tinct. opii, largely diluted with lead water, syringed or dropped into the eye every two or three hours during the remission. During the exacerbation, bathe with moderately cool, pure water, every hour or two, or oftener if pleasant to the eye, either by gently syringing, or by dipping the face into a bowl with the eyes open; repeating the process each time, until the eyes are perfectly cleansed and cooled.

Should this disease progress to the atonic stage, it will require similar treatment with the corresponding stage of purulent opthalmia; which see.

*Muco-purulent, purulent, and gonorrhœal conjunctivitis.*—These diseases are all modifications of that of which I have just treated. Like it, they owe their origin to irritation of the mucous tissue of the conjunctiva; but from it (according to the best authorities) they differ in this, that irritation in those is generally, if not always, caused by infection or contagion, the latter, the gonorrhœal, particularly, resulting only from actual contact of a specific virus, which is capable of infecting only mucous membranes. But there are not wanting eminent authorities, well armed, too, with corroborative facts, who teach that *catarrhal* opthalmia, in vehement and protracted cases, attended with unwonted vascular congestion, followed by muco-purulent, or purulent secretion, becomes contagious, and is so propagated; and even, indeed, that such is often the origin of the Egyptian (purulent) Opthalmia.*

Dr. Veitch remarks, "From whatever cause inflammation of the conjunctiva may originate, when the action is of that nature or degree of violence as to produce a puriform or purulent discharge, the discharge so produced operates as an animal virus when applied to the conjunctiva of a healthy eye."

Most authorities concur with the above, as to general distinction of origin into atmospheric and contagious. But some

---

* See McKenzie, p. 288. Lawrence, p. 162.

contend for the identity of Egyptian opthalmia, and gonorrhœal, and assert that gonorrhœa may be, nay has been produced by application of this discharge from the eye to the urethra.

Purulent conjunctivitis is so variable in intensity, that it is difficult to distinguish the milder cases of it from catarrhal, and the more vehement from gonorrhœal opthalmia. The principal diagnostics from the catarrhal are the great swelling of the palpebræ, and the granular appearance of the thin lining membrane, extreme vascular congestion, deep red color, and chemosis of the conjunctiva, the profuse purulent discharge, and the long continuance of the disease. The stages and periodicity are also less distinctly marked in this, than in the catarrhal.

*Symptoms of the irritable stage.*—A sense of stiffness of the eyelids, and of contraction or tightness of the globe, accompanied with deficiency of mucous secretion, and a sensation as of sand in the eye, with frequent profuse lachrymation characterize this stage, which, when intense, continues only a few hours, in other cases for several weeks. In the latter, the ensuing inflammation commencing at the ciliary margins, slowly progresses on to, and over the globe, followed by muco-purulent, or purulent secretion, and preceded by the extending irritation, which becomes more and more intense, as it advances, from superadded irritation of distention, until the state of the second stage is fully developed.

*Symptoms of the active inflammatory stage.*—Inflammation extended over the ball to the cornea, increased pain, sometimes of excruciating severity, with a sensation as if deep seated in the eye, with violent headache, and throbbing at the temples, and above the eyes, profuse purulent secretion, flowing over the face, and besmearing the bosom ; a sensation as if the eye-ball was too large for its orbit, and as if it would burst, (caused probably by increased secretion of the aqueous humor, from the increased sanguineous determination,) which is sometimes temporarily relieved byactual rupture of the cornea, and discharge of the humor. The disease is now marked by the highest grade of vascular action, with redness and tumefaction of the conjunctiva. The turgid vessels gradually relaxing, fibrine as well as pus begins to be secreted, the membrane becomes thickened and covered with granulations, the eye-lids become red and swollen, the relaxation increases until the palpebræ become extremely tumid, grow paler and become enverted and œdematous. The pain presently diminishes, and secretion of pus and appearance of granulations increase until the third or atonic stage is presented. Throughout this and the preceding stages, well marked remissions and exacerbations occur, that are often periodical, but less regularly so than in catarrhal opthalmia. Constitutional affection is slight, appetite and digestion but little impaired, pulse and tongue generally natural.

*Symptoms of the passive inflammatory stage.*—This stage is characterized by dark red, sluggish, distended vessels in the conjunctiva, great relaxation and chemosis of the membrane, with dark red, sometimes a livid granulated surface, resembling the surface of a ripe mulberry; with nebulæ, ulceration or mortification of the cornea, prolapsus iridis, and staphyloma, sometimes with iritis, cataract, retinitis, leucoma, amaurosis, and atrophy of the eyeball. Or again, in milder cases properly treated, vascular congestion diminishes, pain subsides, swelling recedes, granulations are absorbed, cornea heals or clears up, and the disease resolves.

This is in some cases a most obstinate disease, though generally tractable, and under good management but little dangerous. But without treatment, or under mal-treatment, it becomes terribly destructive ; as illustrated by its effects on the crew of the slave ship Rodeur, recorded by Mr. McKenzie, which, to exhibit the importance of attention to treatment, I subjoin: "The slaves first became affected. In a short time the disease extended to the crew twenty-two in number, of whom, twelve men lost their sight ; one of these was the surgeon ; five lost each one eye ; one of these was the captain ; four had considerable specks and adhesions of the iris to the cornea."* The origin of this disease could not be traced to contagion.

*Treatment of the irritable stage.*—The object of treatment in this stage, is to counteract, and to supersede a specific local irritation of a mucous surface ; and to prevent the occurrence of inflammation, or to mitigate its severity. 1st. By reducing vascular action. 2d. By removing all obstacles to a free and easy circulation of the blood. 3d. By dividing, to lessen intensity of irritation by irritating a greater extent of the mucous surface. 4th. By counter-irritating, or producing another, and if practicable a stronger action in the diseased locality.

The best authorities, with but very few exceptions, concur in recommending very copious general bloodletting in the *commencement* of this disease ; to an extent unparalleled in reference to any other disease ; generally extending it *ad deliquium animi*, and to repeat the operation, until *vascular congestion* is removed or directly diminished, and all pain is relieved. The reason alleged for this unparalleled depletion, in treating a disease described as but little affecting general arterial action, is to "arrest the violent inflammation of the

---

* McKenzie, p. 291.

conjunctiva, to prevent its extension from that membrane to the cornea."

Notwithstanding the imposing character of authorities, imposing on account of their erudition, high order of talent, and above all, on account of their experience derived from the treatment of thousands of cases, neither their facts, their reasoning, nor their objects, appear to me sufficient grounds upon which to establish a course of treatment so illy adapted to the indications in the disease.

To follow in the wake of high authorities, in the treatment of an inflammatory disease purely local; but little if at all disturbing general arterial action, to the shedding of blood to the amount of from thirty to sixty ounces, or to syncope, and repeat *ad libitum*, (none of them speak of less ;) I could no more think of, for their reasons alleged, than of following equally high, and more ancient surgical authorities, in inserting tents, syndons and canulas, into all sorts of wounds, as a necessary part of their dressing. That an increased quantity of blood is always, in natural action, determined to an irritated point, and in proportion to the intensity of irritation, is a surgical axiom, is a fixed law of nature ; and no general depletion, short of an excess, atonic and ruinous, can arrest it.* For whilst there is a sufficiency of blood left in the body to maintain its nervous powers, this supernatural determination to the irritated point, will be maintained.

Purgatives and sudorifics are of more decided efficacy: by determining to the intestines, mesentery, and skin, and by removing impediments to free portal and capillary circulation, they deplete the eye, without debilitating the system ; and

---

* A failure of this effect, when irritation is vehement eventuates in spasms, tetanus and death.

their effect upon the mucous coat of the intestines, contributes temporarily to the third indication of diffusion.

The close analogy that exists between the virus that engenders this disease, and the gonorrhœal; and the well attested efficacy of balsamics and other diffusible stimulants, applied to the mucous surface of the alimentary canal, as counter-irritants in early stages of gonorrhœa, would seem forcibly to admonish us of the importance of recourse to similar treatment in the early stage of this, as well as of gonorrhœal opthalmia. Under the influence of such views, I have in early periods, administered such remedies with decided advantage; of which class, the following mixture is a favorite prescription:

℞. Bals. copaiv. ʒij.

Spt. nit. ether. ʒj.

Spt. tereb. ʒj.

Tinct. op. ʒj.

Gum. acac. ʒij.

Aq. ferv. ℥iij. M. ft. solut.

A tea-spoon full to be taken every four or five hours.

*Local treatment.*—Undiluted tinct. opium poured into the eye two or three times a day—to be applied during remissions only, as irritation subsides, dilute the laudanum with lead water. Bathe with moderately cold water, either by syringing, or by dipping the face into it with the eyes kept open frequently through the day; especially during exacerbations. Where inflammation supervenes about the tarsi, to arrest its further progress, and to prevent agglutination of the margins, keep the palpebræ anointed with some alterative sorbefacient. The following is recommended as such:

℞. Sub-mur. hyd. ʒii.
Acet. plumbi ℥iii.
Axung sui ℥ss. M. ft. Unguentum.

*General treatment of the active inflammatory stage.*—In this stage, increased vigilance is required to keep arterial action below the healthy standard, and to keep the bowels freely open with cooling purgatives. From antimonial sudorifics much benefit is to be expected; not only as aiding to keep down vascular action, and equalize circulation, but to effect counter-irritation throughout a continuous and an associative tissue. All balsamics and stimulants should be discontinued, or administered only during remissions, which, owing to the prolongation of the exacerbations, are often scarcely appreciable in this stage.

*Local treatment.*—Repeated bathings of the eyes and face in moderately cold water, by syringing and by dipping the face into it, with the eyes kept open. Cold water as often as is pleasant to the patient, should be poured over the head and back of the neck. As often as the palpebræ become tumid and swollen, they should be reduced by application of leeches, or by puncturing the most turgid vessels over their surface. Much benefit is sometimes derived from division of prominent vessels on the surface of the globe. If indications of iritis, such as change in its color, or unusual intolerance of light; or of acute cornitis occur, in the progress of this stage, cut off the communication with those by the section of the conjunctiva.

The collyrium to which I am most partial in this stage, is a solution of acet. plumb. in vin. op., from two to six grains to the ounce, to be injected under the lids, or poured into the eye four or five times a day—the eyes having been first cleansed of matter with water.

During the height of the exacerbation, when acute pain, or the sensation of sand in the eye is experienced, much relief and real benefit are derived from the frequent application of the mucilage of the quince seeds, of sassafras pith, or of flax seed. It is but seldom admissible to use any active or stimulating wash where the eyes are thus sensitive and painful; but plasters of opium, of stramonium, or of belladonna, may be beneficially applied around them. The application of the ointment to the palpebræ, prescribed for the first stage, may be beneficially continued during this.

*Treatment of the passive inflammatory stage.*—This is literally the period of disorganization. A greatly increased quantity of blood having been for sometime determined to the delicate vessels of the conjunctiva, and impelled into them with unusual force, their calibres have become dilated, their tunics attenuated, and their contractility lessened, until they remain the mere receptacles of blood, impelled into them by the *vis a tergo;* which still propels a supernatural flow to the part, now laboring under the irritation of disorganization. The capillaries that extend to the cornea, and those that inosculate between the conjunctiva and the ciliary body, presently dilate, and convey red blood instead of colorless lymph. The stimulus of this foreign matter, together with the stimulus of distention, is productive of internal irritation; which elicits an increased flow of blood to the part through the vessels of the choroides and the retina, and becomes productive of inflammatory action in the ciliary body, which rapidly extends to the contiguous membranes, and is frequently followed by amaurosis; either in consequence of disorganization of the retina; or from pressure upon the nerve made by dilatation of the arteria centralis retinæ and the opthalmic branches.

The object of treatment then in this stage, is materially different from that in the first and second. Here it must mainly be directed to restore vascular power in the part, and to change, or to arrest the morbid determination.

*General treatment.*—Tonics, stimulants, and alterants, are the classes of remedies most requisite in treating this stage. In the selection of tonics, especial care should be taken to avoid all such as are known to determine to the head; on this account, quinine and those tonics that contain it should be rejected. The salts of iron, and of bismuth, appear to me to be as unexceptionable as any others. Opium, stramonium, belladonna, hyosciamus, lactucarium, &c., are often of considerable benefit; they lessen irritability, equalize circulation, and allay that exhausting restlessness which is commonly attendant. When distention of inosculating vessels has extended irritation to the ciliary body, and has produced congestion and inflammatory action in the vessels of the choroides and retina, evinced by discoloration of the iris, or misty vision from lenticular obscurity, the use of mercury prosecuted to ptyalism, is the principal, and our most rational dependance. For such in this stage is the atony of the system, that general depletion is not admissible, and if local bleeding were our means of thus depleting, the turgid internal vessels are too limited to be made available. Our only resource then, is to change or divert the morbid determination. Mercury, by the irritation it produces in glandular structures, when brought into active operation, produces an increased sanguineous flow to all such parts, to a degree that depletes a non-secreting portion of body more effectively, than would the abstraction of pounds of blood.

*Local treatment.*—Astringent washes are manifestly best adapted to this stage; alum water, of the strength of from four to ten grains to the ounce, is among the best. The free use of pure spring water will here be beneficial on account of its tonic, as well as of its cleansing and refrigerant effects. No preparation of lead is now admissible either in the form of wash or of ointment. Its sedative, I might say, its paralysing local effect, would increase the inertia, the passive state of the part. The strength of washes should be weakened as diseased action subsides; a solution of alum, or of lead, of full strength, say four to five grains to the ounce, will be borne with but little pain, and with perceptible relief and benefit, if applied to an inflamed eye; but would produce considerable irritation, inflammation and pain, if applied to the healthy conjunctiva.

For this disease of the eyes, but without reference to stage and but little to condition, strong solutions of nit. argent. sulph. cup. per chlor. hydrarg., and of sulphuric, nitric, and acetic acids, have been recommended by high authority. I have used most of them, but never with benefit. Local bloodletting is often highly beneficial. By removing the congestion, action is excited in the sluggish vessels, which is of even more consequence than the depletion. The turgid vessels on the surface of swollen palpebræ, should be often emptied, by puncturing them, or by the application of leeches. Still greater benefit is derived from scarification of the conjunctiva, or division of its vessels on the surface of the eyeball.

In cases of considerable chemosis, Mr. Lawrence recommends radiated incisions to be made through the tumid duplicature of the membrane that projects over the cornea, all around its circumference, to discharge the effusion or ecchymosis from beneath.

The practice of Scarpa, in such cases, of removing with scissors a circular portion of the membrane all around the cornea, is necessarily an inferior operation to the above, as it can effect only the removal of ecchymosis. For from the condition of the part, and the mode of operating, a portion of the mucous coat only is removed; destroying just so much lubricating surface, and leaving the fibrous, the most vascular, the inner coat entire.

Mr. McKenzie's method I think preferable to either. "The mode which I adopt" says he "is to raise a small fold of the conjunctiva with the forceps, and snip it away with the scissors. This fold rarely contains the enlarged vessel which we wish to cut across, but it is now exposed, with a small hook it is easily raised from the surface of the sclerotica and divided."

It is singular, the process in such operations should never have suggested to Mr. McKenzie, the double structure of the conjunctiva.

But in cases in which the transparency or organization of the cornea, or the healthy condition of the internal tunics and humors is endangered, by the violence or the extension to them of the external inflammation, I prefer, to either of those, the simple division of the conjunctiva clear around the cornea, cutting through both of its coats, with all their vessels and nerves. This operation is perfectly conservative in its effects.

Besides emptying the vessels of the conjunctiva, and producing powerful and most benficial alterative action in this membrane, it arrests the further progress and extent of the disease. It is restricted in its action, thenceforth, to the space between the line of section and the tarsi palpebræ, for a period of time, amply sufficient for the accomplishment of a cure of the conjunctivitis.

*The operation.*—To make the section of the conjunctiva, the operator should be furnished with scissors, having a slender probe point, extending a little longer than the extremity of the antagonizing blade; and a common tenaculum. The upper eyelid being raised by an assistant, he should take a secure hold of the conjunctiva with the tenaculum towards either canthus of the eye, near to the edge of the cornea; raise and perforate the coat sufficiently to admit the probe point of the scissors, and at once, and without ever withdrawing the inserted point, divide the membrane entirely around the lower hemisphere, within from one eighth to one fourth of an inch of the margin of the cornea—steadying and turning the eye the while, suitably to the progress of the operation, with the tenaculum held in his other hand. So much having been performed, if the patient can bear more without resting, commence again at the same point, without removing the tenaculum, and proceed to make the section of the upper hemisphere, to intersect that of the lower.

When this operation is completed, the divided edges retract from one eighth to one fourth of an inch, and exhibit the tendinous expansions, the albuginia, through the interstice. But in the course of a few weeks, the adhesive process repairs the breach, and it becomes difficult to detect the cicatrix; nor does there remain any appreciable functional injury.

Care should be taken in making this operation, that the entire conjunctiva is divided through both its coats. In cases of chemosis, which consists in occurrence of effusion between its mucous and fibrous coats, raising and separating the former from the latter, a careless operator would cut none but the former, leaving the fibrous, the most vascular and nervous of the two, entire. By this nothing would be effected but relief from ecchymosis, which would have been better and more easily done by Mr. Lawrence's mode of scarifying.

The treatment after the operation should be just such as though the disease had not so far advanced as to involve or endanger the cornea or the internal structures. The superiority of the operation, in fact, consists only in releasing *those* from any farther liabilities from the conjunctivitis. Should any internal disorganization, or inflammatory action that existed at or before the operation, manifest symptoms of continuance thereafter, it would be safest to subject it to appropriate treatment by mercury, &c. as above advised.

After having practised the above operation nearly twenty years, I met with the following notice of an operation practised in Persia, in the 74th number of the Medico-Chirurgical Review, for the year 1838. It is thus described:

"The object of this operation seems to be, to completely cut off the vascular communication, by excision of a circular portion of the conjunctiva at a small distance from the margin of the cornea, which is accomplished by fixing eight small hooks into the conjunctiva, about a line from the union of the cornea with the sclerotica, quite round the cornea; the operator then raises that part of the conjunctiva by pulling these hooks towards him, and, with a pair of scissors, he cuts off the portion thus raised, and completely insulates the conjunctiva covering the cornea, the consequence of which is the gradual absorption of the opacity of the part affected, and the cornea recovers its transparency. The after-treatment is very simple, consisting merely in the introduction of a small quantity of antimony between the lids; in fact, the result of the operation is confidently expected to be successful without any other application."

I am at a loss to determine whether this is more similar to Scarpa's or to my operation. The effect *ascribed* surpasses either; for it would seem to be radical: I consider mine only conservative. But the mode of operating would indicate that it is the same with Scarpa's; for it is but seldom that a hook raises both coats together, (in chemosis scarcely ever;) and when raised, if the raised part is snipped off, with the flat side of the scissors laid to the eye, as seems to be the mode here, from this description, the fibrous coat with its vessels would generally be left entire beneath, as represented above by Mr. McKenzie, in his mode of scarifying. But the Persian mode is objectionable, (even if it did comprehend both coats,) on account of the unnecessary removal of a portion of the membrane, whereby permanent injury is inflicted; for so broad a cicatrix never can become so perfectly organized as the marginal adhesion of a simple section.

In my operation, the difficulty of passing on the point of the scissors *between* the coats, except in cases of chemosis, and the thinness of the membrane raised, will soon admonish an informed operator of his error, and the facility gained by getting under the fibrous coat, gives assurance that he is right.

I first performed this operation during my residence in Danville, Lincoln county, Ky., in 1819, on Col. D., aet. sixty-eight years, a continental officer of the revolutionary army. I found him afflicted with purulent opthalmia of the right eye, attended with excruciating pain and total blindness. The whole conjunctiva was of a florid red, presenting an extreme degree of vascular congestion; the entire cornea being nebulous and opaque. He complained of deep-seated pain in the ball of the eye, extending over the forehead and to the temples. His left eye was staphylomatous and atrophied, which was represented to have occurred twelve months before from a similar attack. The origin of the disease I could get no satisfactory account of, but ascribed it mainly to intemperance. His appetite was good, tongue slightly furred, pulse hard and frequent. I bled him freely from the arm, cupped the temples and forehead, scarified the conjunctiva, prescribed mercurial purgatives, and sulph. zinc and acet. plumb. solution as a collyrium. Visited him next day; found the congestion increased. Bleeding repeated; small purgatives of calomel ordered nightly.

From this time I repeated my visits every three or four days, a distance of twelve miles; tried several of the most approved collyria of the day, and as taught by the best authorities, applied them without any reference to stage or periodicity. Bloodletting, general and local, and purgatives, were repeated, and abstemious diet rigidly enjoined; until diarrhœa and delirium tremens supervened. Those unpleasant occurrences I had got nearly relieved, when, on the 2d November, having discovered no amendment of the eye, but, on the contrary, increased and increasing thickening and congestion of the membrane, but little relief from pain, and progressive increase of constitutional debility; and having particularly remarked that the enlarged red vessels seen in the nebulous cornea were continuous from the conjunctiva, and that the most prominent of them, when divided in scarifications of the conjunctiva, contracted and gradually disappeared, and that others near those, that were before invisible developed, and were by the next visit frequently found as large and as red as the first had been; I determined to end this alternation and substitution, by dividing them all at once, cutting through the conjunctiva, clear around the cornea. This operation I performed, in this case, with common dissecting forceps and crooked pocket-book scissors. I thereafter prescribed lead-water collyria through the day, and a bread poultice, mixed up with the same, during the night. I remained with him four or five hours after the operation; within that time pain was nearly removed, and inflammation and congestion were very much lessened. On my next visit, the nebulæ of the cornea appeared relaxed and corrugated. On the 10th it hung loose by filaments, and I removed it with forceps, leaving the cornea quite clear. I now considered the eye cured and took leave of the case, although slight congestion of the conjunctiva, and nervous tremors and diarrhœa, still remained somewhat troublesome, leaving a prescription of opium and acet. plumb. for the latter, and a mild collyrium for the former.

On the 16th I was called to see him again, and found him a raving maniac. I advised a more nutricious diet, and the following prescription, with no favorable prognosis:

℞ Sem. stramon. grs. iv.
Cal. grs. ii.
Op. gr. i.

To be given three times a day, the quantity of stramonium to be increased in each successive dose until it dilated the pupil of the eye to its greatest dimensions, and to keep it so dilated until the raving ceased; then gradually to be lessened until sanity was restored. I visited him no more as a patient; but under this course of treatment he recovered in about three weeks, and had remained healthy and in the enjoyment of as good vision as is ordinarily enjoyed at his time of life, up to the time of my removal to Virginia, in May 1820, and I heard of his continued health several years afterwards.

The cases of purulent conjunctivitis with which I have met have generally so readily yielded to treatment, that I have found it necessary to perform this operation in but a single other case. This occurred in the Louisville Marine Hospital, in December last, the subject a native of Ireland, of intemtemperate habits, and was attended with considerable success. The case was treated jointly by Professor Flint and myself, and as he delivered clinical lectures in the Hospital, and prescribed in accordance with the best authorities of the day, I forbore, as a general rule, to interfere with his treatment. The section of the conjunctiva commenced on the 22d and 24th of December, was not completed on account of the obstacles of a remarkably small palpebral fissure, and the want of a hearty disposition to the operation on the part of the patient. The further prosecution of the operation was delayed until late in February, in consequence of indisposition which kept me away from the Hospital. On the 27th of that month the section was completed, and on the next day the inflammation was materially lessened. The case continued to improve, and by the 10th of March, with good general health, only a slight opacity of the cornea remained.

*Gonorrhœal Conjunctivitis.*—This disease is believed in some cases to arise from metastasis from the urethra; but in all cases that I have met with, it seemed to have originated from actual application of gonorrhœal matter to the eye. In its symptoms, it varies not from purlulent opthalmia, except in intensity, and in the rapidity and destructiveness of its progress.

The diagnosis between the milder cases of gonorrhœal and the more violent ones of purulent conjuctivitis, is difficult—is perhaps to be inferred only from the origin, or history of the case. This gonorrhœa like gonorrhœa urethræ, presents various grades of intensity; but in most of its grades, under ordinary treatment, it generally results in loss of vision. The danger to vision is owing only in part, to its specific virus; for it is productive of iritis, retinitis, cataract, and amaurosis, diseases in structures that are not subject to gonorrhœal irritation, they not being mucous tissues; and it is believed that mucous tissues alone, are liable to gonorrhœal irritation, as appears in gonorrhea urethræ. In this, we see the glans penis, the prepuce, and the skin of the thigh, constantly soaked with the discharge in its greatest degree of virulence, without ever becoming involved in the disease. The first application of the contagious matter, indeed, is to the glans, prepuce, and skin of the penis; yet the urethra alone imbibes and takes on the disease. Those internal diseases of the eye then, I conceive should be ascribed not to irritation from gonorrhœal virus, but as an effect of irritation, through sympathy, or I would prefer to say, from irritation by dilitation of inosculating capillaries, by *vis a tergo*. The cornea, is invested with a mucous coat, and is liable to both—irritation from the specific virus, and the irritation from distention; thence it is found sooner and oftener suffering, than any of the other parts important to vision. Of fourteen cases, related by Mr. Lawrence, loss of vision took place in nine "from sloughing, suppuration or opacity of the cornea." "Sight was restored in the other five, with partial opacity of the cornea, and anterior adhesion of the iris in three of the number." "So short a period" says he, "intervenes between the commencement, and the full development of the complaint, that in many instances irreparable mischief is done to the eye, before our assistance is required."

Dr. Physick in his lectures of 1817–'18, said, "that he never had known a cure effected in a case of unquestionable gonorrhœal opthalmia; and that he considered blindness an inevitable consequence." The recollection of this prognosis determined me after my operation on Col. D., that should I ever encounter such a disease, I would embrace the earliest opportunity to make trial of the same expedient in its treatment.

### TREATMENT.

No difference in treatment is required from that recommended in purulent opthalmia. All authorities concur in treating them similarly, or only varying in energy, in proportion to variation of intensity. Some of the most respectable authorities, indeed, consider it probable, that gonorrhœa, had its *origin* through inoculation from Egyptian (purulent) opthalmia.

I have never been consulted in a case of gonorrhœal opthalmia in its early or irritable stage: but from the advantages derived, from the mode above recommended in the treatment of purulent opthalmia in its irritable stage, which it may be seen, is pretty much the same with the ordinary practice in the early stages of gonorrhœa urethræ, with which this is identical, I am disposed to believe, that under similar treatment, gonorrhœal opthalmia, might be, as often cut short, as is gonorrhœa urethræ.

*Case* 1*st.*—A few years after my removal to Fincastle, Virginia, I was called to attend Mr. L., aged twenty-five years, who had a violent purulent opthalmia, which he ascribed to cold. I had his case under vigorous ordinary treatment about two weeks without appreciable amendment; when he revealed a fact, that he had so far not considered worth naming, viz: that prior to my attendance, he had gonorrhœa and that he had several times during the disease, washed his eyes with his urine. This changed the aspect of the case, and I at once apprised him on Dr. Physick's authority of the tendency of his disease, and advised a resort to the circular section of the conjunctiva, as an experiment. He declined it. A few days afterwards he went to Salem, and placed himself under the care of Drs. Gunn and Foot, where he remained until he was perfectly blind ; in which condition he returned and requested me to make trial of the operation I had proposed.

At this time, March 10, 1826, the conjunctiva exhibited the highest degree of congestion in both eyes. The nebulous state of the right eye completely destroyed the transparency of the cornea, over which the vessels interlocked, extending across from opposite sides. The cornea of the left was somewhat opaque, but retained sufficient transparency to exhibit contact of the iris with the cornea, and the opaque lens in contact with, and filling the pupil, the cornea was prominent in the centre, presenting incipient staphyloma. I performed the *sectio conjunctivæ* on the right eye, and treated both with injections of solution of alum, of the strength of grains vi. to the ounce of water. Visited again on the 13th, pain was so much relieved in this eye, that he requested the same operation on the left, *for relief from pain alone*, without hope, promise, or prospect, of restoration of vision.

The same collyrium was continued ; bowels kept open with saline purgatives, diet unirritating but nutritious. After this he complained of but little pain. In twelve days when I quit my regular visits, inflammation had nearly subsided ; the left eye gradually atrophied and flattened ; but was no more painful or troublesome.

The pseudo-membrane of the right eye was not detached, as I had expected, and as had occurred in Col. D's. case ; but was gradually removed by process of absorption ; and about three months elapsed, before the internal condition of the eye was revealed ; when it exhibited immobility of the iris, contraction of the pupil, and cataract.

As soon as I conceived the transparency of the cornea to be sufficiently restored to afford a fair prospect of relief from the operation, I depressed the lens. The operation was speedily recovered from, and without the occurrence of an untoward symptom. The pupil was restored to entire clearness, but without any kind of benefit to vision.

This case although unsuccessfully treated, seemed to me to be of sufficient pathological importance to entitle it to be reported. Here, from a disease arising from a virus that contaminates only by contact, local in its character, and restricted in its locality to mucous tissues, all the important internal structures of the eye have been disorganized. It has produced iritis, synechia anterior, closure of the pupil, cataract, amaurosis, staphyloma, and atrophy. It has deranged and disorganized structures that are not supposed to be susceptible to irritation from gonorrhœal inoculation; and if not, that could only have been acted upon through sympathy, from nervous connection; or by irritation from *vascular* dilatation of insoculating vessels between them and the conjunctiva.

*Case 2d.*—During Mr. L.'s confinement, a healthy, respectable and pious young woman in the neighborhood took great interest in his case. She conceived his opthalmia to have arisen from irritation of wild hairs, and volunteered to pluck them out; and whilst engaged in plucking such of the ciliæ as she conceived to be wild, she, with her fingers, often brushed the sympathetic tear from her own eyes. I was, ere long, called to visit her; found her laboring under severe purulent opthalmia, accompanied with decided iritis; evinced by great intolerance of light in both eyes, and, in one of them, by change of color in the iris. On being informed of the above facts in the history of this case, I could entertain but little doubt of its nature; nor did I hesitate in adopting a vigorous course of antiphlogistic treatment, a minute detail of which is unnecessary—suffice to say, it consisted in bloodlettings, general and local, purging, mercurializing to ptyalism, and in collyria of solutions of acet. plumb., sulph. cup., alum, &c. The treatment had the effect to lessen pain and intolerance of light, to remove discoloration of the iris, and to keep the conjunctivitis pretty much at a stand for nearly two months; during the latter part of which time the conjunctiva cornea gradually thickened, until vision was destroyed in the left eye, and so much obscured in the right as to be of but little utility.

In this state of the disease I performed the section of the conjunctiva on the right, on the 4th of June, 1826; and on the left, on the 7th. After-treatment consisted in the use of occasional mild laxatives of sulph. magnes. and nit. potas., of lead-water collyria through the day, and a poultice containing lead and opium at night.

The film of the left eye was detached from the cornea by interstitial secretion, and thrown off as a pseudo-membrane; and this worst eye was first cured. Transparency was restored to the right by the slower process of absorption. A psorophthalmia, that was troublesome after other symptoms had disappeared, was treated and removed by an ointment of nitrate of mercury and acetate of lead, applied nightly to the margins of the eyelids.

*Case 3d.*—A negro, at James' river Forge, fourteen miles from Fincastle, in May, 1831, got a speck of cinder struck into his eye, and suffered it to remain until it excited inflammation. To relieve this, he prepared a collyrium of zinc and lead, and injected it frequently with a syringe that another workman was using upon himself for a gonorrhœa. Six days after this treatment I was requested to visit him, and found him affected with violent *active* conjunctivitis, complaining of deep-seated pain in the eye, and a sensation as if the ball would burst. Indications of iritis and cornitis were also perceptible; the latter had assumed a greyish cast, and red vessels were seen traversing it from different directions. The iris had assumed a dingy reddish color, in spots about its margin, and a remarkably painful intolerance of light was complained of.

I took from the arm about forty ounces of blood, performed the section of the conjunctiva, prescribed leeches to the palpebræ, daily purgatives of sulph. soda and nit. potas., with the continuation of the collyrium that he had been using, changing the syringe; I visited him no more. About four weeks afterwards he called on me in town with his eye restored.

*Case 4th.*—In September, 1832, a one-eyed laborer at a Furnace twenty-four miles from Fincastle, who had gonorrhœa, became affected with soreness or weakness of the eye. To cure it he washed it with his urine, and eight days after this treatment I visited him. Symptoms as in the above case, iritis excepted. Treatment the same. I never saw him afterwards, but was told that he got well in a fortnight.

*Case 5th.*—A young gentleman who had gonorrhœa, in Fincastle, in 1834, was troubled with a weak eye, which he washed with his urine.* About four weeks afterwards his attending physician requested me to see him for the purpose of operating on the eye, in which he was quite blind. Chemosis was considerable, the cornea transparent but inflamed, and had ruptured near the lower edge; the iris pressed forward and seemed in contact with the cornea; through it was to be seen what we supposed to be the lens, in a state of cataract, but which the sequel induced me to believe was more

* A popular treatment for weak eyes in that region.

probably an adventitious membrane in the pupil. This case had been treated as well as usual in the ordinary way. My prognosis was unfavorable; I gave no promise of restoration of vision, nor any other prospect of benefit from an operation, but to prevent further progress of disorganization and to allay pain. I made the section, prescribed saline laxatives, collyria of lead water, and an unguent of calomel and lead to the palpebræ. In a few days inflammation subsided, and the cornea healed, leaving considerable leucoma. He removed, seven or eight weeks afterwards, out of the neighborhood, at which time he conceived his vision to have improved. I advised him to continue the use of the ointment perseveringly. I was informed several months afterwards, by his friends, that the cataract had disappeared, and that vision was restored.

I have performed the section in other diseases of the conjunctiva, of anomalous or complex character, and have effected restoration from internal degenerations, by medicinal application to that membrane and to the palpebræ, predicated upon the pathological connections heretofore demonstrated—cases that I conceive to be of considerable pathological and surgical interest; but which could be more appropriately connected with a separate dissertation on some of those diseases, than with this, which is, without them, already much longer than I had anticipated or intended.

*April,* 1840.

